# Protective Effects of Melatonin on the Skin: Future Perspectives

**DOI:** 10.3390/ijms20194948

**Published:** 2019-10-08

**Authors:** Iryna Rusanova, Laura Martínez-Ruiz, Javier Florido, César Rodríguez-Santana, Ana Guerra-Librero, Darío Acuña-Castroviejo, Germaine Escames

**Affiliations:** 1 Biomedical Research Center, Health Sciences Technology Park, University of Granada, 18016 Granada, Spain; iryna.rusanova@gmail.com (I.R.); lauramartinezruiz8@gmail.com (L.M.-R.); javi.florido@hotmail.com (J.F.); cesar@correo.ugr.es (C.R.-S.); ana.guerralibrero@gmail.com (A.G.-L.); dacuna@ugr.es (D.A.-C.); 2 Department of Physiology, University of Granada, 18016 Granada, Spain; 3 Centro de Investigación Biomédica en Red de Fragilidad y Envejecimiento Saludable, Instituto de Investigación Biosanitaria CIBERFES, IBS. Granada, Granada Hospital Complex, 18016 Granada, Spain

**Keywords:** skin, melatonin, mitochondria, oxidative stress, aging

## Abstract

When exposed to hostile environments such as radiation, physical injuries, chemicals, pollution, and microorganisms, the skin requires protective chemical molecules and pathways. Melatonin, a highly conserved ancient molecule, plays a crucial role in the maintenance of skin. As human skin has functional melatonin receptors and also acts as a complete system that is capable of producing and regulating melatonin synthesis, melatonin is a promising candidate for its maintenance and protection. Below, we review the studies of new metabolic pathways involved in the protective functions of melatonin in dermal cells. We also discuss the advantages of the topical use of melatonin for therapeutic purposes and skin protection. In our view, endogenous intracutaneous melatonin production, together with topically-applied exogenous melatonin and its metabolites, represent two of the most potent defense systems against external damage to the skin.

## 1. Introduction

Skin constitutes an essential defense against a wide variety of environmental toxins in order to protect the internal organs. Its strategic location as a key barrier between the external environment and the internal milieu renders skin a crucial instrument for preserving body homeostasis [1]. However, skin often shows signs of degeneration that contributes to the aging phenotype and numerous pathological processes [2].

Melatonin, which is a potent free radical scavenger with antioxidant and anti-inflammatory activity and an ability to maintain mitochondrial homeostasis under different experimental conditions, contains powerful anti-aging properties [3]. Several studies have revealed that mammalian skin possesses a melatoninergic system [4,5], where melatonin synthesis decreases with aging. However, topical melatonin has been shown to improve the clinical signs of skin aging [6], promote skin wound healing [7,8,9], and to treat many skin pathologies such as atopic dermatitis [10,11,12,13], seborrheic dermatitis [14], and vitiligo [15,16]. Thus, the pleiotropic biochemical action of melatonin at the skin level could represent an effective anti-aging strategy as well as an excellent therapeutic treatment for skin pathologies.

In our view, melatonin is a very important molecule for protecting and maintaining cutaneous homeostasis and for mitigating the clinical signs of skin aging.

## 2. Melatonin as Skin Cellular Protector

Melatonin (N-acetyl-5-methoxytryptamine, aMT), a highly conserved molecule widely found in nature, was first isolated and chemically identified in bovine pineal tissue by Lerner et al. in 1958 [17]. The circadian production of melatonin by the pineal gland explains its chronobiotic impact on organismal activity including endocrine and non-endocrine rhythms [18]. However, following the identification of melatonin synthesis in the pineal gland, melatonin-related enzymes have been detected in many other peripheral tissues and organs such as the skin [19]. Thus, melatonin is a molecule that regulates circadian day–night rhythms and seasonal biorhythms [20,21], and is also a multifunctional hormone that performs numerous functions to protect the organism from environmental changes. In this regard, given that the skin acts as a protective barrier, melatonin plays a particularly important role.

## 3. Synthesis and Metabolism of Melatonin in the Skin

The concentration of melatonin in the skin is several times higher than that in plasma [22]. Studies have revealed that mammalian skin possesses a fully functioning melatoninergic system [5,19,23]. This system not only includes the two principal enzymes involved in melatonin synthesis, aralkylamine N-acetyltransferase (AANAT) and acetylserotonin O-methyltransferase (ASMT), but also in gene expression, protein synthesis, and enzymatic activity. However, intermediates of melatonin synthesis such as hydroxytryptophan can also be generated through the free radical oxidation of L-tryptophan. It has been suggested that environmental factors such as solar radiation may also be involved in regulating the skin melatoninergic system.

Slominski et al. discovered that melatonin and its metabolites in the human epidermis depend on race, gender, and age. Interestingly, the highest concentration of melatonin has been found in the epidermis of African-Americans [4,22]. According to the classification of phototypes of skin color based on the ability to tan and the tendency to burn, these individuals have skin type 3 or 4 and less risk of skin cancer [24]. Melatonin is metabolized in the skin through the indolic pathway and produces metabolites such as 6-hydroxymelatonin and 5-methoxytryptamine. However, melatonin can also be metabolized to N1-acetyl-N2-formyl-5-methoxykynuramine (AFMK) through the kynuric pathway, which involves both enzymatic and nonenzymatic processes carried out by ultraviolet radiation (UVR) and free radicals [4,25,26]. Ultraviolet B (UVB) can also induce the metabolism of AFMK to N1-acetyl-5-methoxykynuramine (AMK) [27]. Interestingly, as they are more potent than melatonin itself in reducing oxidative stress, the metabolites AMK and AFMK can play an important role in photoprotection.

## 4. Mechanism of Action of Melatonin in the Skin

The action of melatonin on the skin resembles that of other peripheral tissues and organs. Melatonin, which is highly lipophilic, crosses all biological barriers, affects all subcellular compartments including mitochondria and nuclei, and can act directly or through receptors.

### 4.1. Melatonin Receptors in the Skin

Two groups of melatonin receptors have been identified and characterized: membrane receptors and nuclear receptors [28]. Membrane receptors, type 1 (MT1) and type 2 (MT2), bind high-affinity melatonin [29] and are coupled to the G protein [30]. However, the low affinity melatonin receptor type 3 (MT3), characterized by low melatonin affinity and located in the cytosol of some cells, acts independently of the G protein [31].

Genes encoding the MT1 receptor have been detected in epidermal locations such as hair follicle keratinocytes and dermal papilla fibroblasts [32], while MT2 is predominantly found in adnexal structures such as eccrine glands, the inner root sheath, and the blood vessels of human skin [33,34,35], although very low levels of these receptors have been observed in melanocytes [36]. Although little is known about the presence of the MT3 regulator in human skin, according to Nosjean et al., it is similar to the regulatory protein quinine reductase 2 (NQO2) [37], which is involved in the antioxidant and antiapoptotic functions of melatonin in the skin.

On the other hand, the nuclear receptor RORα has been identified in adult epidermal keratinocytes, fibroblasts, and hair follicles as well as in melanocytes and several melanoma cell lines [38,39,40,41]. Pineal melatonin is well known to interact with RORs, which act as important transcription factors and mediate many biological consequences of melatonin including anti-inflammatory and anti-oxidative effects, although less is known about the specific impact of RORs on the skin. Dai et al. identified the powerful anti-inflammatory effects of a synthetic RORα/γ inverse agonist on mouse models of atopic dermatitis and acute irritant dermatitis [42]. However, further research is needed to identify the molecular mechanisms involved in the binding of melatonin to these receptors.

### 4.2. Receptor-Independent Functions of Melatonin

Despite the existence of the melatonin receptor, many of the actions of melatonin are mediated through receptor-independent activities such as antioxidation and anti-inflammation as well as mitochondrial activity.

### 4.3. Melatonin as a Potent Antioxidant

Melatonin is a potent free radical scavenger that is capable of neutralizing reactive species by donating an electron. Unlike classic antioxidants, melatonin is devoid of prooxidative activity, while all known intermediates generated by the interaction of melatonin with reactive species are free radical scavengers defined as the free radical scavenging cascade reaction of the melatonin family [43]. Due to this cascade, one melatonin molecule has the capacity to scavenge up to four or more reactive species [44]. Melatonin can also indirectly affect the skin through its metabolites, which act as potent antioxidants. For example, hydroxyl derivatives of melatonin and AFMK metabolites, which are greater antioxidants than melatonin itself, are produced in response to ultraviolet (UV) stimulation [45]. Moreover, in skin cells constantly exposed to external aggressors, melatonin can bind reactive oxygen species (ROS) and inhibit their generation by activating cytosolic flavoprotein quinone reductase II (NQO2) [46]. Therefore, the direct scavenging of reactive oxygen species (ROS) by melatonin plays an important role in the skin by attenuating UVR-induced oxidative damage. New antioxidant mechanisms of melatonin have recently been described by Janjetovic et al. (2017), who demonstrated that melatonin activates the expression of nuclear factor erythroid 2-related factor 2 (Nrf2) to protect keratinocytes against UVR-mediated oxidative stress [36]. Due to the exposure of cells to ROS, Nrf2 can be translocated to the nucleus and activate gene expression of a series of antioxidative and cytoprotective proteins including heme oxygenase-1 (HO-1), NAD(P)H dehydrogenase, quinone 1 (NQO1), γ-glutamyl-cystein synthase (γ-GCS), glutathione peroxidase (GPx), glutathionine S-transferase (GST), glutathione reductase (GRd), and superoxide dismutase (SOD) [47].

In other words, the presence of melatonin in the skin is of paramount importance in its protection against damage caused by external factors.

### 4.4. Melatonin as a Potent Mitochondrial Protector

Melatonin’s ability to cross through cell membranes and to reach cellular organelles such as mitochondria is highly important for maintaining mitochondrial homeostasis in different pathological situations [48,49]. Mitochondria are powerful organelles required for ATP synthesis and/or heat production and play an important role in Ca^2+^ homeostasis, in the production of free radicals such as reactive nitrogen and oxygen species (RNS/ROS), which act as cell signaling messengers, and also in activating the NLRP3 inflammasome. Mitochondrial compartments contain the highest cellular concentrations of melatonin, where melatonin synthesis and metabolism take place [50]. Moreover, melatonin metabolites (AFMK, 5MT and 6-hydroxymelatonin) can be accumulated in the human epidermis both in vivo and in vitro in response to ultraviolet radiation (UVR), thereby contributing to mitochondrial energy production [36]. Many studies have focused on the importance of maintaining the mitochondrial membrane potential for generating ATP and correct mitochondrial function. As an antioxidant protector of cardilipin and mitochondrial permeability transition pores (MPTP), melatonin increases mitochondrial membrane potential and ATP production, enhances the activity of electron transport chain (ETC) complexes [51,52,53], and thus maintains mitochondrial efficiency.

Melatonin also plays an active role in mitochondrial homeostasis by regulating biogenesis and mitophagy. Recent studies have suggested that it induces protein expression, which regulates autophagic events and mitochondrial dynamics, and restores mitochondrial function by removing damaged mitochondria through a process of mitophagy [54].

### 4.5. Melatonin as a Potent Anti-Inflammatory Agent

The inhibition of the NF-κB pathway explains many of the anti-inflammatory functions of melatonin. A recent study by Park et al. (2018) demonstrated the protective effects of melatonin on UVR-treated human keratinocytes by reducing NF-κB expression, following an increase caused by UVR [55]. Thus, melatonin is a widespread anti-inflammatory molecule capable of inhibiting the expression of numerous inflammatory markers such as inducible nitric oxide synthase (iNOS)/i-mtNOS and COX-2, and pro-inflammatory cytokines such as TNF-α under various pathophysiological conditions [48,56]. However, ROS-generating mitochondria and the NLRP3 inflammasome have been reported to be connected [57]. Mitochondrial oxidative damage activates NLRP3, resulting in the release of proinflammatory interleukins (ILs) such as IL-1β and IL-18 and the activation of certain pro-apoptotic pathways [58]. In addition to inhibiting the NF-κB pathway, topical application of melatonin inhibits NLRP3 and mitochondrial dysfunction [57], thereby attenuating inflammatory cytokines. Thus, as UVR induces inflammation and mitochondrial disruption, which are involved in chronic oxidative stress, melatonin has been shown to ameliorate DNA damage by inhibiting the NF-κB and NLRP3 pathways [56,57].

**Figure 1:**
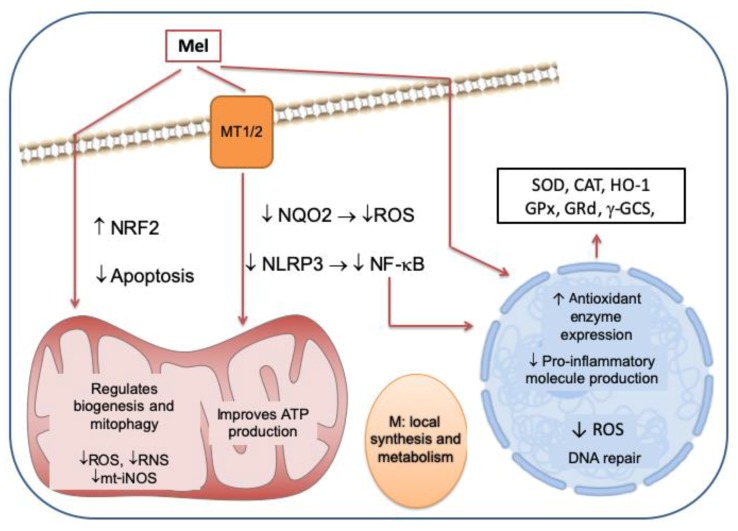
Melatonin crosses cell membranes and acts through membrane (MT1/2) and nuclear (ROR) receptors in order to reach different cellular organelles including mitochondria and nuclei. Melatonin’s antioxidant properties are characterized by its capacity to stimulate the production of antioxidant enzymes such as superoxide dismutase (SOD), catalase (CAT), heme oxygenase-1 (HO-1), glutathione peroxidase (GPx), glutathione reductase (GRd), and γ-glutamyl-cystein synthase (γ-GCS). Its antioxidant and cytoprotective properties are boosted by its ability to induce the expression of NRF2 and to bind and inhibit reactive oxygen species (ROS) through NQO2 in the cytosol. Melatonin can also inhibit NLRP3 inflammasome and reduce NF-κB expression, leading to a reduction in proinflammatory molecules. Melatonin ameliorates DNA damage induced by external factors and has an antiapoptotic effect. At the mitochondrial level, it directly scavenges ROS and inhibits m-iNOS expression, which neutralizes both reactive oxygen and nitrogen species (ROS/RNS) in mitochondria, which improves oxidative phosphorylation and ATP production. Melatonin is synthesized and metabolized in mitochondria (M) and also plays an active role in mitochondrial homeostasis by regulating biogenesis and mitophagy. Therefore, it plays a major role in protecting the skin against damage caused by external factors.

The protective functions of melatonin described above are summarized in Figure 1.

## 5. Functions of Melatonin in the Skin

### 5.1. Melatonin as a Photoprotector

Although UV has beneficial effects on human skin by increasing vitamin D and endorphins, excessive exposure entails serious health risks including erythema, atrophy, pigmentary changes, wrinkling, and malignancy. Photodamage can lead to the development of photoaging and cancer in chronically exposed skin [59].

**Figure 2:**
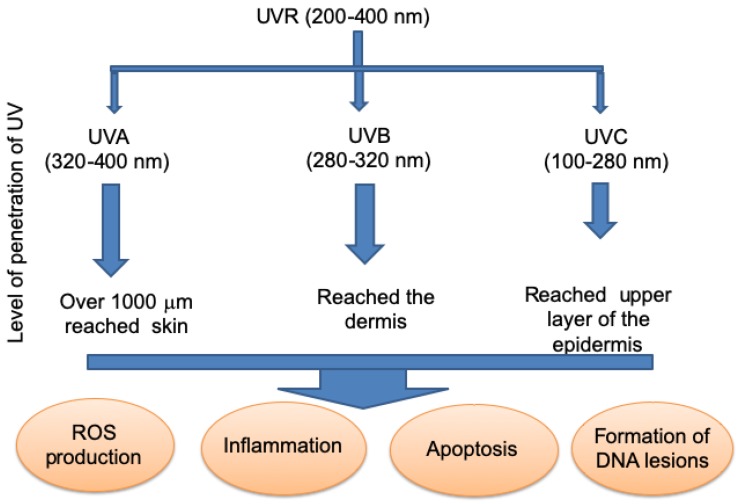
Ultraviolet radiation (UVR) with wavelengths between 200–400 nm is categorized into UVA, UVB, and UVC and can damage human skin. UVA penetrates the underlying sub-cutaneous tissues, UVB can reach the dermis, while UVC can only penetrate the upper layer of the epidermis. The exposure of the human skin to natural ultraviolet radiation (especially UVB) or artificially-generated UVR, produces damage caused by the production of reactive oxygen species (ROS), inflammatory processes, accelerated apoptosis, and the formation of DNA photo lesions.

UVR, along with visible light and infra-red rays, is one of the constituents of the solar spectrum. Ultraviolet A (UVA) radiation penetrates deeply into the dermis, while UVB penetrates the epidermis, with little reaching the dermis [60]. However, UVC has difficulty penetrating the atmosphere and can reach the upper layer of the epidermis (Figure 2). When the skin is exposed to natural sunlight or to artificially generated UVR, it is damaged by the generation of ROS, inflammatory processes, accelerated apoptosis, and the formation of DNA photo lesions [61,62] (Figure 2). In addition to free radical formation, UV directly affects DNA nucleotide base pairing [63]. Shorter wavelength UV photons, particularly UVB and UVC, cleave internal pyrimidine double bonds, which are particularly vulnerable to chemical alteration by the absorption of UVR energy, leading to highly mutagenic photoproducts and dimers in the skin [36,63]. UV from sunlight is therefore the principal etiological agent of skin cancer, which is a serious health problem worldwide.

Scientists began to study the pre- and post-irradiation effects of melatonin on skin cells two decades ago. In 2001, Ryoo et al. reported that melatonin significantly prevented damage associated with UVB irradiation in cultured dermal fibroblasts [64]. The increased expression of the melatonin membrane receptors, MT1 and MT2, following skin cell irradiation also highlights the important role of melatonin in irradiated cells [39,65]. Studies by Lee et al. [66] and Ranieri et al. [67] showed that keratinocytes exposed to H_2_O_2_ present molecular damage similar to that caused by UVA. Treatment of these cells with melatonin to enhance the autophagy process is a primary mechanism in protecting keratinocytes against hydrogen peroxide-induced cell damage through sirt1 activation. In addition, sirtuin 1 (Sirt1) has an anti-inflammatory and cytoprotective effect through the downregulation of PI3K/Akt, MAPKs, and NF-κB signaling [68]. A recent study by Janjetovic et al. revealed that irradiation of cultured human melanocytes with UVB stimulates ROS production, which was reduced by treatment with melatonin or its metabolites such as AFMK [36]. They also demonstrated that Nrf2 plays an important role in the protective action of melatonin and its metabolites, which, in line with previous studies, also repairs DNA in melanocytes exposed to UVB [65]. In 2018, Skobowiat et al. [41] used human and porcine ex vivo skin to evaluate the effects of melatonin and its active derivatives on UVB-induced damage. Porcine skin, which is similar to human skin, is an excellent model for biomedical research. The topical application of melatonin and AFMK protects epidermal cells against UVB-induced DNA damage and apoptosis [41]. Moreover, the photo protective effect of melatonin was apparent both before and after UVB treatment and was more pronounced when skin was treated with melatonin and its metabolites prior to UVB exposure. Clinical studies of humans have shown that the application of melatonin cream protects against natural sunlight-induced erythema [69]. A sunscreen formulation fortified with melatonin has a superior sun protection factor and the ability to counteract ROS.

Thus, melatonin and its metabolites protect skin cells including keratinocytes [70], melanocytes [36], fibroblasts [64], and leukocytes [71] from UV-induced damage. The exogenous application of melatonin and its derivatives constitutes a powerful promising tool to prevent UV-induced oxidative stress and DNA damage. The literature on these issues is summarized in Table 1.

### 5.2. Melatonin as a Radioprotector

Continued exposure to radiation during repeated diagnostic medical imaging, radiotherapy, or chemotherapy can cause damage to the skin. A 2001 study found that melatonin decreases apoptotic cell populations in x-ray irradiated cultured skin fibroblasts [72]. Another study in 2005 reported data on the capacity of melatonin to minimize the injurious effects of x-ray on the skin [73]. Intraperitoneal injection of melatonin minimizes signs of cellular damage in different skin cells including the destruction of epidermal cells, swollen mitochondria, and intracellular changes. A recent study evaluated the protective effects of melatonin on gamma ray-induced skin damage in Wistar rats and demonstrated an amelioration of skin damage at the molecular and histopathological level. Malondialdehyde (MDA), inflammation, and hair follicle atrophy levels were found to decrease, while antioxidant enzyme (CAT and SOD) activity was observed to increase following the administration of melatonin, thus highlighting its radioprotective effects [74]. To evaluate the radioprotective properties of melatonin-based creams in humans, Ben-David et al. carried out a prospective, randomized, placebo-controlled, double-blind study of phase II in patients who underwent breast-conserving surgery for stage 0–2 breast cancer and received radiation therapy. Radiation dermatitis, one of the complications of radiotherapy, was found to be significantly lower in the melatonin group when compared to patients receiving the placebo [75].

The mechanisms involved in melatonin used as a radioprotective agent have been attributed to its capacity to reduce oxidative damage through effective free radical scavenging, especially at the mitochondrial level. It also increases antioxidant enzyme expression and activity, which reduces lipid peroxidation and DNA damage caused by irradiation [56,76], and inhibits inflammasome, leading to an attenuation of inflammatory processes [57].

Other molecular pathways are also involved in the radioprotector function of melatonin, which decreases apoptosis by inhibiting p53 and Bax and by increasing the antiapoptotic protein Bcl-2 [65,77]; it also increases Nrf2 expression [36] and Sirt1 [66] involved in repairing lesions in cellular DNA [77]. In 2015, Ortiz et al. demonstrated that melatonin gel reduced and prevented oral mucositis induced by irradiation [56]. Although their study was carried out at the level of oral mucosa, the results could also apply to skin cells, which have a similar high proliferative capacity.

### 5.3. Melatonin as a Protector against Skin Damage

Melatonin is a molecule that can break through lipid skin barriers, reduce oxidative stress, modify mitochondria function, reduce inflammation, and affect the expression of certain genes. Consequently, melatonin has excellent beneficial antiaging effects on the skin, whose visible expression is a reduction in wrinkle formation.

The regenerative potential of tissue declines with aging due to changes in the proliferation and differentiation of stem cells [78]. In addition, damage induced by oxidative stress and inflammation at the molecular level results in a decrease in collagen production, while the epidermis becomes thinner and less immune to external aggressors. It is well documented that aged or wrinkled skin presents elastic fiber and collagen degradation and elasticity loss [79]. The photoaging process is initiated by an increase in ROS production, leading to an activation of the NF-κB pathway [80]. This induces the expression of pro-inflammatory factors [81,82], leading to an increase in inflammatory skin processes and the activation of proteases such as matrix metalloproteinases (MMPs), which weaken the structure of the skin. A gradual increase in MMP expression has been proposed as one of the mechanisms that alter the elastic fiber network. The elastase activity of these enzymes is present in the dermis in response to repetitive UV exposure [79]. Sung-Hoon Kim studied the anti-wrinkle mechanism of melatonin in keratinocytes and hairless mice [55] and found that melatonin suppresses ROS production and, consequently, reduces MMP-1 expression and increases collagen XVII expression in keratinocytes treated with UVB. Melatonin has also been shown to significantly reduce water loss on the dorsal skin of hairless mice eight weeks after UVB treatment, while water loss was accentuated in the control treatment with UVB alone when compared to the normal untreated control [55].

Additionally, ROS induces the activation of the activator protein-1 (AP-1) and Hedgehog (HH) pathway, which control a number of cellular processes including differentiation, proliferation, and apoptosis. For example, the zinc finger protein GLI1, also known as glioma-associated oncogene, is a transcription factor and one of the nuclear executors at the end of the HH pathway responsible for regulating downstream target genes and is involved in epidermal development during organogenesis, homeostasis, epidermal repair, and hair follicle development [55,83]. However, aberrant activation of HH signaling is associated with various skin tumors [84].

**Figure 3:**
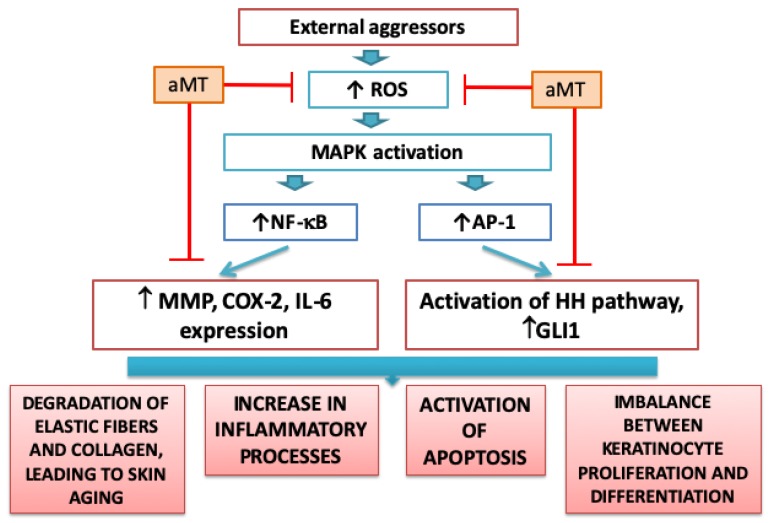
External aggressors increase ROS production in the skin, which induces downstream MAPK signaling pathways, leading to an activation of NF-κB and AP-1. Activation of the NF-κB pathway induces the expression of MMPs, COX-2, and IL-6, among others. This leads to an increase in inflammatory processes and activates proteases that weaken the structure of the skin. Activation of the HH pathway through the involvement of GLI1 increases apoptosis and leads to an imbalance between keratinocyte proliferation and differentiation.

The mechanisms involved in skin damage induced by external aggressors as well as the protective actions of melatonin are summarized in Figure 3.

One of the factors that influences the aging of skin is the absence of estrogen during menopause. Studies of humans and animals show that lack of estrogen in the skin is associated with decreased thickness of the epidermis and dermis, wrinkling, dryness, atrophy, decreased collagen, elasticity loss, and poor hair growth [85,86]. Uslu et al., who studied the effects of melatonin treatment on the skin of postmenopausal rats [87], found that melatonin, at a dosage of 30 mg/kg/day intraperitoneally administered for four weeks, increased the epidermal thickness, the subcutaneous fat layer, and elastic fibers when compared to the controls. They observed an increase in the fibroblast growth factor-β (FGF-β), collagen type I, and fibronectin as well as high c-Myc and β-catenin expression following melatonin treatment of the epidermis and hair bulb in both normal and ovariectomy mice [87]. Previous studies have shown that c-Myc expression in fibroblasts can induce pluripotent stem cells in mouse skin [88] and is involved in reprogramming fibroblasts into induced pluripotent stem cells in different pathological situations [89,90]. In vitro studies have suggested that β-catenin is involved in positively regulating fibroblast proliferation during the wound healing process [91].

Atopic dermatitis (AD) is a chronic multifactorial inflammatory skin disorder, with a high prevalence in children, accompanied by sleep disturbance and deteriorating nighttime pruritus [92]. Higher nighttime melatonin levels have been reported to be associated with reduced sleep disturbance and milder AD symptoms in children [93]. Several studies have reported that melatonin ameliorates inflammatory parameters such as serum C-reactive protein (CRP) levels [94,95] as well as IL-4 and IFN-γ production [96]. Studies by Chang et al. of 48 children [93] and by Ardakani et al. of 70 patients with AD [97] found that melatonin administered orally may affect AD by decreasing wake time at night, reducing scratching events, and by modulating certain immune system parameters. Both studies reported decreased total serum IgE levels, while melatonin supplementation significantly improved the scoring atopic dermatitis index and total sleep time. The oral administration of melatonin, with low doses of 3 mg over four weeks in the Chang study and of 6 mg over six weeks in the Ardakani study, may boost the benefits of AD treatment with melatonin.

As melatonin levels are low in the blood with oral administration due to marked first-pass degradation in the liver, its access to the skin is limited and should therefore be administered topically in clinical dermatology [35]. Most studies have used orally administered melatonin, which can significantly reduce its efficiency as the amount reaching the skin through this type of administration is quite limited. Although these studies demonstrate the effects of melatonin’s mechanisms of action, very high levels of melatonin need to be administered orally to be effective. It is therefore necessary to explore the implications of the topical administration of melatonin in order to circumvent this problem.

## 6. Topical Application of Melatonin

Ethanol solutions and cream applied topically have been used to investigate the systemic bioavailability and percutaneous penetration of melatonin [98,99]. Both formulas were found to increase serum levels for 8 h [98] and 24 h [99] hours following application, depending on the protocol used in the experiment. This indicates that melatonin is able to penetrate the skin and accumulate in the stratum corneum through prolonged release over a 24 h period from the skin into the blood system [35]. It would also be worthwhile to explore new topical melatonin formulations that do not reach the blood.

Some studies of the topical use of melatonin for different purposes are described below.

Sierra et al. studied various emulsions containing 1% melatonin combined with a mixture of three ultraviolet filters (MMIX) in order to find a topical formulation of melatonin to protect the skin against the harmful effects of UV radiation [100]. In addition to the good results for in vivo skin protection against UV exposure damage, 80% of the medication was released from the emulsion within 2 h, with a high penetration coefficient [100].This interacts with the lipid membrane, which facilitates the penetration of melatonin, probably due to the lipophilic properties of the three UV filters.

To evaluate their antiaging efficacy in the skin, Milani M. and Sparavigna A. used day and night creams containing melatonin vehiculated in lipospheres (Melatosphere) [6]. Their results showed that these topical melatonin formulations improved the hydration and tonicity of the aging skin of women when compared to the non-treated control skin, with a significant reduction in skin roughness and a clinical improvement in the appearance of wrinkles. Therefore, endogenous intracutaneous melatonin production, together with melatonin and its exogenous metabolites applied topically, appears to be a powerful antioxidant defense strategy against skin damage induced by UV rays and an effective antiaging technique.

The topical application of melatonin can also be used to treat alopecia [101]. A recent study revealed that melatonin, combined with nanostructured lipid carriers (NLCs), exhibits high entrapment efficiency and anti-oxidant potential in addition to sustained release lasting 6 h. Furthermore, the NLCs displayed good storage stability and increased the skin deposition of melatonin 4.5-fold in the stratum corneum, 7-fold in the epidermis, and 6.8-fold in the dermis when compared to the melatonin solution. Melatonin NLCs recorded more clinically desirable results when compared to the melatonin solution in patients with androgenic alopecia and increased hair density and thickness, while decreasing hair loss [101].

## 7. Future Perspectives

The topical application and transepidermal delivery of natural melatonin are attractive strategies for dermatologists and for maintaining healthy skin in general. As seen throughout this review, skin, with its own fully functioning melatoninergic system, can synthesize and metabolize melatonin, whose metabolites such as AMK and AFMK, are more effective than melatonin itself in reducing oxidative stress and may play an important role in photoprotection. However, the production of melatonin decreases with age and skin remains the organ most exposed to environmental stressors including radiation. Melatonin molecules in the pineal gland regulate the circadian day–night rhythm and seasonal biorhythms, while melatonin tissue is a multifunctional hormone that performs numerous functions that protect the organism from environmental changes.

Melatonin can cross cell membranes to reach different cellular organelles and acts, directly or through receptors, in all subcellular compartments including mitochondria and nuclei. Its anti-oxidant properties enable melatonin to scavenge ROS, which are mostly produced in mitochondria, and to stimulate the production of antioxidant enzymes. Its anti-inflammatory properties enable it to inhibit NLRP3 inflammasome and reduce NF-κB expression, leading to a reduction in pro-inflammatory molecules. At the mitochondrial level, its protective properties enable melatonin to ameliorate ATP production, regulate mitochondrial homeostasis, inhibit apoptosis, repair the epidermis, and enhance hair follicle growth. All these properties point to the major role played by melatonin in protecting the skin against damage caused by external factors. Moreover, the photoaging process is initiated by increased ROS production, which induces the expression of MMPs and others molecules, leading to an increase in inflammatory skin processes and an activation of proteases that weaken the structure of the skin.

Melatonin circulating in the blood has limited access to the skin due to significant first-pass degradation in the liver, a problem that can be circumvented by its topical administration. Unlike oral administration, topically applied melatonin is considered as effective protection against the harmful effects of UV radiation. It is also recommended for the treatment of alopecia and atopic dermatitis and to ameliorate skin tonicity, leading to a significant reduction in skin roughness and wrinkles. Important studies have recently been carried out on new skin protection techniques and time-dependent aspects of skin barrier functions as well as skin chronopharmacology. Finally, the use of topically applied melatonin is a very promising area of preventive skin medicine.

## Figures and Tables

**Table 1:** Studies of the effects of melatonin on irradiated skin cells.

Type of Cell	Melatonin Dosage	Effects	Authors
Human keratinocytes (HaCaT)	Cells were preincubated with melatonin at graded concentrations from 10^−9^ to 10^−3^ M for 30 min prior to UV irradiation at doses of 25 and 50 mJ/cm^2^.	Pretreatment with melatonin inhibited apoptosis, increasing DNA synthesis, and number of colonies.	[71]
Human keratinocytes (HaCaT) and normal human epidermal keratinocytes (NHEK)	Before UVR, cells were pre-incubated for 1 h with melatonin (10^-3^ M) and irradiated with increasing UVR doses (0, 10, 25, 50 mJ/cm^2^).	At 48 h post-UVR, melatonin effectively protected cells, decreased disturbances in plasma membrane potential and changed intracellular pH, caused by irradiation (25 or 50 mJ/cm^2^). The presence of melatonin significantly protected the cells -12% (HaCaT) and 14% (NHEK)	[72]
Ex vivo full human skin thickness	Skin was preincubated with melatonin (10^−3^ M) and exposed to UVR in a dose- (0, 100, 300 mJ/cm^2^) and time-dependent manner (0, 24, 48 h post UVR).	Pre-incubation of skin samples with melatonin led to significant reductions in 8-OHdG-positive cells and prevention and depletion of antioxidative enzymes (CAT, GPx, Cu/ZnSOD, MnSOD).	[73]
Human full-thickness skin in organ culture and cultured normal human epidermal keratinocytes (NHEK)	Human skin and cells were preincubated with melatonin (10^−3^ M) and exposed to UVR in a dose (0, 100, 300 mJ/cm^2^)- and time-dependent manner (0, 24, 48 h post-UVR).	Melatonin inverted the increase in *Hsp70* gene expression and Hsp70 protein levels in skin, as well as the decrease in enhanced gene expression of pro-inflammatory cytokines (*IL-1b, IL-6, Casp-1*) and pro-apoptotic protein (*Casp-3*) in NHEK.	[74]
Human keratinocytes (HaCaT)	Cells were exposed to formulations with 1% *w*/*v* melatonin solutions and controls for 2 h and then irradiated with a single dose of UVB (26 mJ/cm^2^).	Reduced generation of ROS and lower caspase 3 and 7 enzymes activities in cells previously treated with melatonin.	[62]

## Abbreviations

5MT	5-methoxytryptamine
AANAT	Aralkylamine N-acetyltransferase
AD	Atopic dermatitis
AFMK	N1-acetyl-N2-formyl-5-methoxykynuramine
AMK	N1-acetyl-5-methoxykynuramine
aMT	N-acetyl-5-methoxytryptamine, melatonin
AP-1	Activator protein-1
ASMT	Acetylserotonin O-methyltransferase
CAT	Catalase
CRP	C-reactive porotein
COX-2	Cyclooxygenase-2
ETC	Electron transport chain
FGF-β	Fibroblast growth factor-β
GLI1	Glioma-associated oncogene transcription factor
GPx	Glutathione peroxidase
GRd	Glutathione reductase
GST	Glutathionine S-transferase
HH	Hedgehog pathway
HO-1	Heme oxygenase-1
IFN-γ	Interferon type II (IFN-γ in humans)
ILs	Interleukins
iNOS	Inducible nitric oxide synthase
MAPKs	Mitogen-activated protein kinases
MDA	Malondialdehyde
MMPs	Metalloproteinases
MPTP	Mitochondrial permeability transition pores
MT1	Membrane receptors type I
MT2	Membrane receptors type II
MT3	Membrane receptors type III
mtNOS	Mitochondrial nitric oxide synthase
NF-κB	Nuclear factor kappa-light-chain-enhancer of activated B cells
NLCs	Nanostructured lipid carriers
NQO1	NAD(P)H dehydrogenase [quinone] 1
NQO2	NAD(P)H dehydrogenase [quinone] 2, flavoprotein quinone oxidoreductase II
Nrf2	Nuclear factor erythroid-2-related factor 2
RNS	Reactive nitrogen species
RORα	Retinoic acid related orphan receptor alfa
ROS	Reactive oxygen species
SOD	Superoxide dismutase
γ-GCS	γ-glutamyl-cystein synthase
